# What effect do attempts to lose weight have on the observed relationship between nutrition behaviors and body mass index among adolescents?

**DOI:** 10.1186/1479-5868-4-40

**Published:** 2007-09-19

**Authors:** Jennifer Utter, Robert Scragg, Cliona Ni Mhurchu, David Schaaf

**Affiliations:** 1 Epidemiology and Biostatistics, School of Population Health, University of Auckland, Auckland, New Zealand; 2 Clinical Trials Research Unit, School of Population Health, University of Auckland, Auckland, New Zealand; 3 Pacific Health, School of Population Health, University of Auckland, Auckland, New Zealand

## Abstract

**Background:**

Little research has given consideration to how people's weight control behaviors may moderate the relationships between nutrition and body mass index (BMI) in large cross-sectional studies. The objective of the current study is to determine how attempts to lose weight confound the relationships between nutrition behaviors and BMI among a population of predominately overweight adolescents.

**Methods:**

Data were drawn from the baseline measurements of the Pacific OPIC (Obesity Prevention In Communities). Participants included approximately 3500 high school students in New Zealand. Students in the sample primarily identified as a Pacific Island ethnicity (57%) and the mean age for participants was 14.8 years. Participants completed a questionnaire about nutrition and physical activity patterns and were weighed and measured for height.

**Results:**

In our sample, 57% of students were overweight/obese, with the highest prevalence among Pacific Island students (71%). Approximately 50% of students were currently trying to lose weight, and this was more common among females, Pacific Island students and overweight/obese students. Examination of the nutritional correlates of BMI in the total population found inverse relationships between BMI and consumption of high-fat/high-sugar foods and positive relationships between BMI and eating 5 or more fruits and vegetables a day (all significant after controlling for age, sex, and ethnicity). For example, students who drank the most soft drinks or ate fruit and vegetables infrequently had the lowest mean BMI. Students' attempts to change their weight significantly moderated the relationships between most nutritional behaviors and BMI. In most cases, among students not trying to change their weight, expected relationships were observed; among students trying to lose weight, unexpected or no relationships were observed.

**Conclusion:**

Our findings suggest that among this population of predominately overweight students, solely relying on cross-sectional findings between nutrition behaviours and BMI would misinform intervention strategies. It appears that many students are already taking appropriate steps to reduce their weight. Intervention efforts should now move beyond education-based strategies to environmental changes that support students in adopting healthier nutrition practices.

## Background

In New Zealand, approximately one third of children aged 5–14 years are overweight or obese[1] and, when compared internationally, it has been estimated that New Zealand has witnessed an annual increase in the prevalence of childhood obesity greater than most other industrialised countries[2]. With the increasing awareness of the health implications of obesity, weight control behaviors are now common among adolescents in New Zealand. More than 60% of girls and 29% of boys are trying to lose weight[3]. International research has documented that weight control behaviors are more common among overweight adolescents [4] and that the most common types of weight control strategies adolescents employ are dietary changes and increasing their physical activity[5].

Little research has given consideration to how people's weight control behaviors may moderate the relationships between nutrition behaviors and body mass index (BMI) in large cross-sectional studies. This is important because nutrition and health surveys often identify the associations between dietary and activity patterns and obesity and these findings form the basis of many intervention strategies and weight loss advice. For example, the positive cross-sectional associations between television viewing and obesity were being reported as early as 1985[6]. Consequently, reducing television viewing became a significant component of several obesity prevention interventions[7,8]. Given the increasing prevalence of obesity and the general awareness of related issues, it is possible that cross-sectional research may be increasingly confounded by the weight control attempts of its population. While it is most likely that unexpected findings go unreported, one study from the Third National Health and Nutrition Examination Survey highlighted that low water consumption and high energy consumption were significantly associated with a healthful weight among adolescents [9]. One possible explanation for this finding may be that overweight students are drinking more water and reducing their caloric intake as a way to control their weight. Without adequate discussion of these unpredicted findings, there may be potentially serious implications for obesity prevention efforts: increased water consumption and reduced caloric intake become reported as significant correlates of overweight and obesity.

Thus, the aim of the current study is to determine the nutritional correlates of obesity (e.g. consumption of breakfast, fruits and vegetables, and high-fat/high-sugar foods) among a predominately overweight population of high school students in New Zealand and to determine how students' attempts to change their weight moderates the cross-sectional relationships between BMI and nutritional behaviors.

## Methods

### Study design

Data for the current study were collected during baseline measurements of the Pacific OPIC (Obesity Prevention in Communities) Project. OPIC is a four country community-based obesity prevention study among predominately Pacific adolescents with sites in Australia, New Zealand, Fiji and Tonga. Data for the current study were drawn from participants at the New Zealand site. Students from seven high schools in South Auckland were invited to participate. These schools were purposively selected for inclusion in the study because they had sufficient numbers of Pacific Island students and were in close proximity to each other. All of the schools participating in the study were classified as decile 1 or 2; school deciles reflect the socioeconomic position of its students and range from 1 (most deprived) to 10 (least deprived).

Students completed a questionnaire about their eating and physical activity patterns on a hand-held computer and were weighed and measured for height. All data were collected at school, during the school day, and in the presence of trained staff between February and October 2005. The University of Auckland Human Participants Ethics Committee granted ethical approval for the study. Active consent was received by students aged 16 years and older and by parents/guardians of students under the age of 16.

### Participants

All students attending school during the days of data collection were invited to participate. Based on the school rolls, it was estimated that 6766 students were eligible for participation. The final study response rate was 54% (n = 3626); information about the non-responders was not available. The study sample was comprised of slightly more females (52%) than males (48%) and had a mean age of 14.8 years (range 12 to 20 years). Students in the final study sample identified their ethnicity primarily as Pacific Island (57%), followed by New Zealand Maori (20%), then European (12%) and Asian/other (11%).

### Measures

#### Sociodemographic characteristics

Students' *ethnicity*, *age *and *gender *were each assessed by self-report. Students were asked which ethnic group they most identified with and were given 11 ethnicities from which to choose. Students were then categorised into four main ethnic groups: 'Pacific' (Samoan, Cook Island Maori, Tongan, Niuean, or Other Pacific), 'Maori', 'European' (NZ European or Other European), and 'Asian/other' (Chinese, Indian, Other).

#### Weight change attempts

*Weight change attempts *were assessed by one question asking students what they were currently trying to do about their weight. Students could respond, 'lose weight', 'gain weight', 'stay the same weight', or 'I'm not trying to do anything about my weight.' The last two categories were combined for analyses and are referred to as 'not change weight'.

#### BMI

Height and weight measurements were taken by trained research staff using standardised protocols. Students wore light clothing and no shoes. Students' weights were measured to the nearest 0.1 kg using a digital scale. Students' heights were measured to the nearest 0.1 cm using a free-standing portable stadiometer. Body mass index (BMI) was calculated as weight (kilograms) divided by height (meters) squared. Weight status (obese, overweight, and normal weight) was defined using international definitions for children and adolescents[10].

#### Nutrition behaviors

The nutrition behaviors chosen for analyses were selected because of the available evidence in New Zealand and internationally, which indicates these behaviors as correlates of overweight and obesity[6,11-15]. *Breakfast consumption *was assessed with the question, 'In the last 5 school days, on how many days did you have something to eat for breakfast before school started?' Students were asked about their usual *fruit and vegetable consumption *separately with the questions, 'How many serves of fruit (vegetables) do you usually eat each day?' *Fast food/takeaway food consumption *was assessed with the question, 'How often do you usually eat food from a takeaway?' (local examples provided). Consumption of *after school snacks *that were high in fat or high in sugar was assessed with two questions about the frequency of eating fried foods or chocolates, sweets, or ice cream after school. *Soft drink consumption *was estimated with two questions: 'In the last 5 school days (including time spent at home), on how many days did you have regular (non-diet) soft drinks?' and 'On the last school day, how many glasses or cans of soft drinks did you have? Average soft drink consumption was estimated by multiplying the number of days soft drinks were consumed by the previous day's consumption and then averaged over the previous five school days. The estimated amounts were then categorised into five categories of approximate quintiles.

### Analysis

The survey procedures in the statistical software SAS (Cary, NC, version 9.1) were used to correct design effects from clustered sampling. The demographic characteristics describing students by their weight status and weight control attempts were generated by cross-tabulations using chi-square tests to determine statistical differences. The analyses describing the demographic characteristics of students by their weight control attempts were conducted stratified by gender because males and females differ in their desires to change their weight[3] Multivariate regression models were used to determine the associations between each nutrition behavior and BMI, while controlling for age, sex, and ethnicity. A separate set of multivariate regression models were used to examine the effect of the interaction between each of the nutrition behaviors and weight change attempts on BMI while controlling for age, sex, and ethnicity. A test was considered to be statistically significant if p < 0.05.

## Results

Nearly 60% of the participating students in the sample were overweight/obese (Table 1). Weight status was not associated with age or gender, but was significantly associated with ethnicity. Pacific Island adolescents were more likely to be overweight/obese (71%), and Asian/other adolescents less likely to be overweight/obese (28%), compared with adolescents of other ethnicities. Nearly half of all students were trying to lose weight, 40% were trying to stay the same weight or were doing nothing about their weight, and 14% were trying to gain weight (Table 2). A higher proportion of females than males were trying to lose weight, similar proportions of males and females were not trying to change their weight, and more males than females were trying to gain weight. Age was associated with attempting to change weight in males but not females. Among males, younger adolescents were more likely to report attempting to lose weight, while older adolescents were more likely to be trying to gain weight. For both genders, ethnicity and weight status were significantly associated with weight change attempts. Pacific Island students and overweight/obese students were more likely to be trying to lose weight.

**Table 1:** Demographic characteristics of study population by weight status.

	**Obese**			**Overweight**			**Normal weight**			
	n	%^1^	SE^2^	n	%	SE	n	%	SE	p-value^3^
Total	926	26.5	2.9	1100	31.5	1.7	1464	41.9	3.8	
										
Gender										
Male	436	26.2	2.6	508	30.5	1.5	719	43.2	3.3	
Female	490	26.8	3.3	592	32.4	2.2	745	40.8	4.5	0.31
										
Age										
12–13 years	230	29.8	1.5	251	32.5	2.5	292	37.8	3.0	
14 years	225	26.3	3.2	277	32.3	3.5	355	41.4	3.4	
15 years	164	23.5	3.8	219	31.4	2.9	314	45.1	6.1	
16 years	159	25.8	3.3	194	31.4	2.2	264	42.8	4.6	
17+ years	148	27.1	5.3	159	29.1	1.5	239	43.8	5.5	0.54
										
Ethnicity										
Pacific Island	690	34.6	1.4	721	36.1	1.2	584	29.3	0.7	
Maori	164	23.1	2.9	212	23.1	2.9	334	47.0	2.3	
Asian/other	26	6.9	2.0	79	20.9	1.5	273	72.2	1.5	
European	46	11.3	4.8	88	21.6	3.3	273	67.1	5.5	<0.001

^1 ^Row percentage, unadjusted for confounders

^2^Standard error (SE) for percentage

^3^P-value for chi-square

**Table 2:** Demographic characteristics of study population by attempts to control their weight.

	**Lose weight**			**Gain weight**			**Not change weight**^1^			
	n	%^2^	SE^3^	n	%	SE	n	%	SE	p-value^4^
Total	1662	47.6	1.4	475	13.6	0.9	1353	38.8	1.6	
										
**MALES**										
Total	657	39.5	1.8	355	21.4	1.4	651	39.2	2.4	
										
Age										
12–13 years	188	50.7	2.6	58	15.6	2.3	125	33.7	2.2	
14 years	177	42.8	2.0	80	19.3	2.7	157	37.9	3.5	
15 years	110	32.5	3.0	73	21.5	4.2	156	46.0	3.5	
16 years	97	32.7	1.8	79	26.6	3.8	121	40.7	3.7	
17+ years	85	35.1	3.1	65	26.9	0.6	92	38.0	3.1	<0.001
										
Ethnicity										
Pacific Island	452	46.5	1.3	204	21.0	1.1	317	32.6	1.1	
Maori	102	32.9	4.1	53	17.1	1.1	155	50.0	3.6	
Asian/other	50	25.4	2.4	69	35.0	3.4	78	39.6	5.4	
European	53	29.0	2.7	29	15.9	1.8	101	55.2	3.1	<0.001
										
Weight Status										
Obese	339	77.8	1.4	5	1.2	0.4	92	21.1	1.6	
Overweight	221	43.5	2.4	73	14.4	1.9	214	42.1	2.5	
Normal weight	97	13.5	1.5	277	38.5	3.4	345	48.0	3.3	<0.001
										
**FEMALES**										
Total	1005	55.0	1.7	120	6.6	0.5	702	38.4	1.5	
										
Age										
12–13 years	210	52.2	3.0	26	6.5	0.8	166	41.3	2.8	
14 years	249	56.2	1.6	35	7.9	1.3	159	35.9	1.2	
15 years	208	58.1	2.2	23	6.4	0.8	127	35.5	2.2	
16 years	174	54.4	3.2	15	4.7	0.5	131	41.0	3.4	
17+ years	164	54.0	4.2	21	6.9	1.2	119	39.1	4.4	0.31
										
Ethnicity										
Pacific Island	613	60.0	1.2	63	6.2	0.8	346	33.9	1.1	
Maori	209	52.3	2.0	24	6.0	0.7	167	41.8	1.7	
Asian/other	80	44.2	3.2	18	10.0	2.5	83	45.9	2.1	
European	103	46.0	4.8	15	6.7	1.3	106	47.3	4.5	<0.001
										
Weight Status										
Obese	386	78.8	1.9	6	1.2	0.6	98	20.0	1.5	
Overweight	392	66.2	1.3	19	3.2	0.6	181	30.6	1.1	
Normal weight	227	30.5	3.3	95	12.8	1.6	423	56.8	2.3	<0.001

^1^Includes students who were trying to stay the same weight or were not trying to do anything about their weight.

^2 ^Row percentage, unadjusted for confounders

^3^Standard error (SE) for percentage

^4^P-value for bivariate relationship

The relationships between each of the nutrition behaviors and BMI for the total sample are displayed in Table 3. Significant inverse relationships between BMI and consumption of breakfast, fast food/takeaways, and high fat or high sugar after school snacks were observed. For example, students who ate breakfast on all of the past five school days, and who ate fast food takeaways most days of the week, had significantly lower mean BMIs than students who never ate breakfast or who ate fast food takeaway less than once per month. Conversely, students who reported eating five or more fruits and vegetables a day had a significantly higher mean BMI (p = 0.002) than students who did not.

**Table 3:** Bivariate and multivariate relationships between BMI and nutrition behaviors in the total sample.

	**Bivariate Relationship**			**Multivariate Relationship**^1^		
	n	Mean BMI^2^	95% CI^3^	β^4^	SE^5^	p-value
Breakfast consumption (days per school week)						
None	689	26.4	(25.1, 27.7)	0.97	0.1	
1–2 days	429	26.9	(25.7, 28.2)	0.96	0.4	
3–4 days	924	25.9	(24.6, 27.3)	0.22	0.2	
5 days	1037	24.8	(23.5, 26.0)	0.00	0.0	<0.001
						
Eat five or more fruits and vegetables a day						
No	2028	25.4	(24.1, 26.7)	-0.78	0.1	
Yes	1458	26.2	(25.0, 27.4)	0.00	0.0	0.001
						
Average daily soft drink consumption						
Not regular drinker	757	25.3	(23.9, 26.7)	0.00	0.0	
1/2 a can a day or less	1053	25.6	(24.3, 27.0)	-0.19	0.1	
1 can a day	655	25.8	(24.5, 27.1)	-0.37	0.2	
2 cans a day	340	26.4	(24.8, 27.9)	0.23	0.3	
More than 2 cans a day	398	26.0	(25.2, 26.8)	-0.48	0.1	0.022
						
Fast food/takeaway consumption						
Once a month or less	694	26.2	(24.4, 27.9)	0.00	0.0	
2–3 times a month	973	25.9	(24.7, 27.1)	-0.52	0.3	
1–3 times a week	1562	25.5	(24.4, 26.6)	-1.25	0.3	
Most days	256	25.5	(24.2, 26.8)	-1.87	0.3	0.002
						
High fat foods as after school snack						
Never	874	25.4	(23.8, 26.9)	0.00	0.0	
Some days	1735	26.0	(24.8, 27.2)	-0.38	0.2	
Most days	633	25.6	(24.5, 26.7)	-1.34	0.2	
Everyday	243	25.4	(24.2, 26.6)	-1.66	0.4	<0.001
						
High sugar foods as after school snack						
Never	744	26.3	(25.0, 27.6)	0.00	0.0	
Some days	1689	25.8	(24.7, 27.0)	-0.98	0.1	
Most days	704	25.1	(23.9, 26.4)	-1.90	0.2	
Everyday	348	25.2	(23.7, 26.8)	-1.80	0.2	<0.001
						
Fruit as after school snack						
Never	480	24.6	(23.4, 25.9)	0.00	0.0	
Some days	1508	25.9	(24.6, 27.1)	0.85	0.3	
Most days	819	26.5	(25.2, 27.8)	1.79	0.4	
Everyday	678	25.3	(23.9, 26.7)	0.98	0.4	0.002

^1 ^Controlling for age, sex, and ethnicity

^2 ^Unadjusted mean

^3 ^95% Confidence Interval (CI) for the mean

^4^Regression coefficient ^5^Standard error (SE)

**Figure 1:**
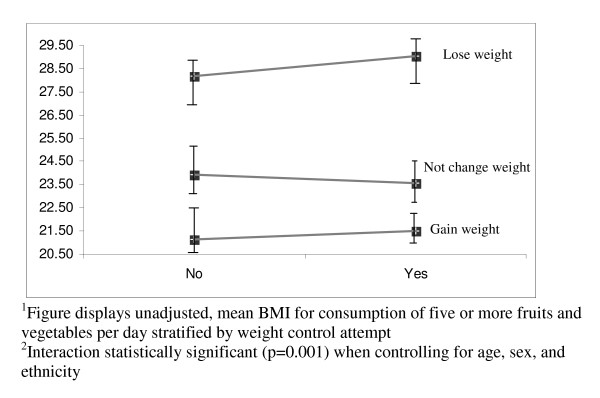
Visual representation^1 ^of the significant interaction^2 ^between consumption of five or more fruits and vegetables a day and weight control attempt on BMI.

**Figure 2:**
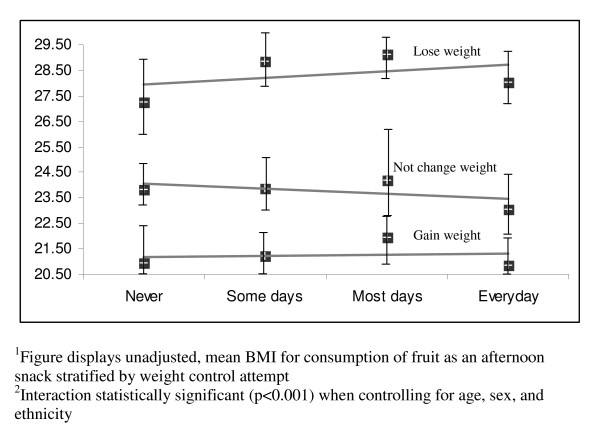
Visual representation^1 ^of the significant interaction^2 ^between consumption of fruit as an afternoon snack and weight control attempt on BMI.

**Figure 3:**
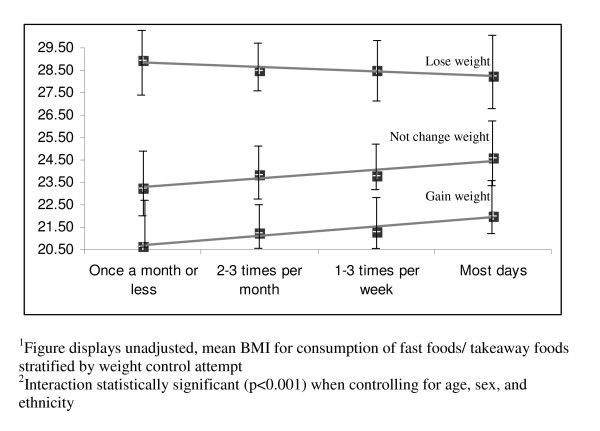
Visual representation^1 ^of the significant interaction^2 ^between consumption of fast food/takeaway foods and weight control attempt on BMI.

**Figure 4:**
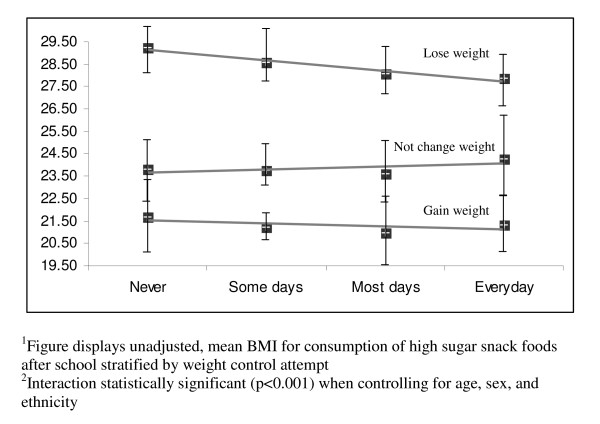
Visual representation^1 ^of the significant interaction^2 ^between consumption of high sugar snack foods after school and weight control attempt on BMI.

Table 4 describes how weight change attempts moderate the relationships between each of the nutrition behaviors and BMI. The p-values reflect the significance of the interaction terms (expressed as nutrition behaviour*weight change attempt) controlling for age, sex, and ethnicity. The mean BMI for each behavior, stratified by weight change attempt, describes the direction of the interaction. In most analyses, weight change attempts significantly moderated the effect of the nutrition behaviors and BMI. The most significant interactions are visually displayed in Figures 1, 2, 3, 4. Among students not trying to change their weight, eating five fruits and vegetables a day (Figure 1) or having fruit as an after school snack (Figure 2) is associated with a lower BMI, while among students trying to lose weight the reverse is true. More frequent consumption of fast food/takeaways (Figure 3) and high sugar snacks after school (Figure 4) is associated with a higher BMI among students not trying to change their weight, but this relationship was not observed for students trying to lose weight. The direction of the relationships between BMI and the nutrition behaviours among the students trying to gain weight were inconsistent.

**Table 4:** Relationships between BMI and nutrition behaviors stratified by weight control attempt.

	**Lose weight**			**Gain weight**			**Not Change weight**^1^			
	n	BMI^2^	95% CI^3^	n	BMI	95% CI	n	BMI	95% CI	p-value^4^
Breakfast consumption (days per school week)										
None	347	28.8	(27.1, 30.5)	76	21.3	(19.9, 22.8)	266	25.0	(23.7, 25.9)	
1–2 days	225	29.4	(28.1, 30.7)	48	22.7	(20.9, 24.4)	156	25.0	(23.4, 25.9)	
3–4 days	425	28.8	(27.4, 30.1)	142	21.3	(20.2, 22.4)	357	24.4	(22.9, 25.8)	
5 days	465	28.1	(26.8, 29.3)	149	20.8	(19.7, 21.8)	423	22.5	(21.7, 23.4)	0.003
										
Eat five or more fruits and vegetables a day										
No	886	28.2	(27.0, 29.4)	294	21.1	(19.7, 22.6)	847	23.9	(22.8, 25.0)	
Yes	773	29.0	(27.7, 30.3)	180	21.5	(21.0, 22.0)	505	23.6	(22.6, 24.6)	0.001
										
Average daily soft drink consumption										
Not regular drinker	394	28.0	(26.5, 29.4)	78	20.5	(19.1, 22.0)	285	22.9	(21.4, 24.3)	
≤1/2 a can a day	518	28.7	(27.4, 29.9)	125	20.6	(19.0, 22.2)	410	23.4	(22.1, 24.6)	
1 can a day	294	28.7	(27.3, 30.0)	94	21.5	(19.8, 23.3)	267	24.1	(23.1, 25.1)	
2 cans a day	148	29.3	(27.2, 31.4)	56	22.5	(21.2, 23.9)	136	24.8	(23.1, 26.5)	
>2 cans a day	169	28.8	(27.3, 30.3)	71	21.6	(20.5, 22.7)	158	25.0	(24.1, 25.8)	0.038
										
Fast food/takeaway consumption										
Once a month or less	388	29.0	(27.4, 30.5)	75	20.6	(18.6, 22.7)	231	23.3	(21.9, 24.6)	
2–3 times/month	495	28.5	(27.5, 29.4)	115	21.3	(20.1, 22.4)	363	23.9	(22.5, 25.3)	
1–3 times/week	674	28.5	(27.0, 30.0)	228	21.3	(20.2, 22.5)	660	23.8	(23.0, 24.7)	
Most days	102	28.3	(26.2, 30.3)	56	22.0	(21.0, 23.0)	98	24.6	(22.8, 26.3)	<0.001
										
High fat foods as after school snack										
Never	456	28.3	(26.9, 29.7)	84	20.2	(18.6, 21.7)	334	22.7	(21.5, 23.9)	
Some days	850	28.8	(27.6, 30.0)	217	21.3	(20.1, 22.4)	668	24.0	(23.2, 24.9)	
Most days	259	28.6	(27.2, 29.9)	117	22.1	(21.3, 22.9)	257	24.2	(22.9, 25.5)	
Everyday	94	28.4	(26.8, 30.0)	56	21.2	(19.6, 22.8)	93	24.9	(23.6, 26.2)	0.001
										
High sugar foods as after school snack										
Never	375	29.2	(28.2, 30.2)	90	21.7	(19.6, 23.7)	279	23.8	(22.5, 25.1)	
Some days	835	28.6	(27.2, 30.0)	214	21.2	(20.7, 21.8)	640	23.8	(22.9, 24.6)	
Most days	306	28.1	(26.9, 29.2)	110	21.0	(19.3, 22.7)	288	23.6	(22.2, 25.0)	
Everyday	143	27.9	(26.4, 29.3)	60	21.3	(20.1, 22.5)	145	24.3	(22.4, 26.1)	<0.001
										
Fruit as after school snack										
Never	185	27.3	(25.6, 28.9)	91	21.0	(19.5, 22.5)	204	23.8	(23.0, 24.7)	
Some days	709	28.9	(27.6, 30.1)	207	21.2	(20.1, 22.4)	592	23.9	(22.8, 24.9)	
Most days	426	29.1	(27.8, 30.4)	98	22.0	(20.7, 23.3)	295	24.2	(22.6, 25.8)	
Everyday	339	28.0	(26.7, 29.4)	78	20.9	(20.0, 21.7)	261	23.1	(21.7, 24.4)	<0.001

^1^Includes students who were trying to stay the same weight or not trying to do anything about their weight.

^2 ^Unadjusted mean

^3^95% Confidence Interval (CI) for the mean

^4^p-value for the interaction term expressed as behavior*weight control attempt, controlling for age, sex, and ethnicity

## Discussion

The aim of the current study is to determine how attempts to change weight moderate the cross-sectional relationships between BMI and nutritional correlates among a predominately overweight population of high school students in New Zealand. In a population with a high percentage of students who are overweight/obese and are trying to lose weight, weight control attempts significantly moderated the relationships between nutrition behaviors and BMI. These findings have significant implications for the interpretation of future cross-sectional studies examining nutritional correlates of BMI and for obesity prevention programs.

The current study population represents a group of adolescents at increased risk for obesity and related health issues. The population was predominately comprised of adolescents of Pacific Island ethnicities and from poorer socioeconomic position and, in New Zealand, childhood obesity rates are highest among these groups [1]. The prevalence of overweight/obesity among Pacific Island children in New Zealand is substantially higher than many other ethnic populations of other western countries [16-18]. In our population of high school students in South Auckland, 59% of students were overweight/obese, with more than 70% of the Pacific students classified as overweight/obese. Furthermore, nearly half of the sample was currently trying to lose weight, indicating an increased need for health advice and support for healthy eating and physical activity for this population.

In analyses of the total sample, previously documented nutritional correlates of obesity were either not significantly associated with BMI or were significant, but in the opposite direction to that expected. These findings are inconsistent with a large body of evidence that support a positive relationship between excess body weight and skipping breakfast[13], soft drink consumption [12,19-21], and eating food prepared away from home[11,14,22], indicating that these behaviors should be targeted by prevention programs. We did find, however, that students' attempts to change their weight significantly moderated the relationships between BMI and consumption of breakfast, fruits and vegetables, soft drinks, fast food and high sugar/high fat afternoon snacks. In most cases, among students who were trying to lose weight, the students with the highest BMI's were eating the fewest unhealthy foods and the most healthy foods. These findings suggest that adolescents who are trying to lose weight may already be adopting the dietary changes targeted by health promotion programs, such as increasing consumption of fruits and vegetables and decreasing consumption of high-fat or high-sugar foods.

It is also possible that significant interactions observed in the current study may reflect a reporting bias whereby overweight adolescents underreported their consumption of high-fat or high-sugar snacks. Research with adults has indicated that underreporting is more common among women and those who are overweight or trying to lose weight[23]. Underreporting energy intake is also common among children and adolescents; 20% of young people underreported their energy intake in the UK National Diet and Nutrition Survey [24]. Among adolescents, underreporting is more common among those who are older [24,25] and overweight [24,26]. Furthermore, there has been some suggestion that measures of dietary restraint do not correspond with actual dietary restriction[27], suggesting that students who report trying to lose weight may be reporting a perceived need to lose weight rather than an actual behavior change. That said, we do not believe it to be likely that underreporting or misreporting of weight control attempts explain all of the significant interactions in our study. The strength and consistency of the interactions across several nutrition behaviors, including the healthier behaviors, and the relevance of these behaviors to health promotion messages suggests that adolescents are trying to make appropriate dietary changes. Our findings are consistent with previous research documenting that dietary changes are among the most common weight control strategies employed by adolescents[5] and that adolescents who are trying to lose weight eat more fruits and vegetables than their peers[28,29]. By either explanation, these findings suggest that further education-based interventions in this population may not be of any additional benefit as these young people appear to know about appropriate dietary strategies for weight control.

The current study is unique in its large, ethnically diverse sample of adolescents at increased risk for obesity, but there are several limitations of our findings that are important to consider. First, because of the uniqueness of the demographic characteristics of our population, generalization of our findings to other countries or other populations with high risk for obesity are limited. Second, the final student response rate was modest at 54%. Because information about the non-responders is not available, we cannot hypothesise how the non-responders may have biased our results. Third, there may be moderators other than attempts to change weight that explain the inconsistent findings between the nutrition behaviours and BMI in the current study. For example, the only indicator of socioeconomic position in the current study was school decile. It is possible that student household income could vary within the schools and that household income could have an independent confounding effect. However, indicators from the 2001 Census of the main intervention area for the OPIC study suggest that this area is relatively economically disadvantaged and there may not be much variation in household socioeconomic position[30] Also ethnicity is highly correlated with socioeconomic indicators in New Zealand [31] and we have controlled for the effects of ethnicity in the current analyses.

## Conclusion

Findings from the current study suggest that attempts to change weight significantly modified the relationships between BMI and nutrition behaviors. This finding is important to consider in future cross-sectional studies of BMI and nutrition behaviors, especially among populations who may be heavily targeted by obesity prevention programs. Future cross-sectional research may need to exclude people who are currently trying to lose weight from analyses examining the relationships between nutrition behaviors and BMI. This approach has been previously used to correct for confounding by smoking in the relationships between BMI and mortality[32]. Future studies examining relationships between BMI and nutrition behaviours that report nonsignificant or unexpected findings should consider that attempts to change weight may be modifying these relationships.

Our findings also suggest that the young people in our study are already adopting healthier behaviors in attempt to control their weight. To improve the effectiveness of obesity prevention programs, efforts may be better targeted to younger children and their families to adopt healthier eating practices before they become overweight since weight loss is difficult to achieve. Adolescents who diet to lose weight are more likely to gain weight into adulthood[33] and are more likely to adopt more extreme dieting behaviors [34]. Environmental changes that support young people in eating healthy foods may improve the effectiveness of existing obesity prevention programs that are primarily education-based by supporting young people to change and maintain their dietary patterns[35].

## Competing interests

The author(s) declare that they have no competing interests.
